# The prevalence and predictive factors of breast cancer screening among older Ghanaian women

**DOI:** 10.1016/j.heliyon.2020.e03838

**Published:** 2020-04-27

**Authors:** Akosua F. Agyemang, Agnes Naki Tei-Muno, Veronica Millicent Dzomeku, Emmanuel Kweku Nakua, Precious Adade Duodu, Henry Ofori Duah, Anna Boakyewaa Bentil, Pascal Agbadi

**Affiliations:** a Department of Social Work, University of Ghana, P.O. Box LG 419, Ghana; b Methodist University College Ghana, Department of Social Sciences, Social Work Unit, P.O. Box DC 940, Dansoman Accra, Ghana; c Department of Nursing, Faculty of Allied Health Sciences, College of Health Sciences, Kwame Nkrumah University of Science and Technology, Kumasi, Ghana; d Department of Epidemiology and Biostatistics, School of Public Health, Kwame Nkrumah University of Science and Technology, Kumasi, Ghana; e Research Department, Foundation of Orthopaedic and Complex Spine Hospital, Accra, Ghana

**Keywords:** Public health, Epidemiology, Women's health, Health education, Aging and life course, Breast cancer screening, Firth logistic regression, Older adult women, Ghana

## Abstract

**Background:**

Breast cancer cases are on the rise in Ghana, with older adult women being more at risk of the disease. However, there is a paucity of current studies on factors that predict breast cancer screening among older adult women using nationally representative data. The present study, therefore, addressed this gap by estimating the prevalence of and identifying the factors that predict breast cancer screening among older adult women in Ghana.

**Methods:**

We used the cross-sectional survey dataset of the 2014/2015 (wave II) Study on global AGEing and adult health (SAGE). A complex survey design methodology was employed to estimate the prevalence of breast cancer screening and the descriptive statistics of the demographic characteristics of the respondents. We used the firth logistic regression for the bivariate and multivariate analysis.

**Results:**

The estimated breast cancer prevalence among older Ghanaian adult women was 4.5%. Older Ghanaian adult women who have screened for cervical cancer [AOR: 13.29; CI: 6.12, 28.84], had attained some primary education [AOR: 3.70; CI: 1.94, 7.07], junior secondary [AOR: 4.02; CI: 1.75, 9.21], senior secondary and higher [AOR: 4.57; CI: 2.15, 9.71], and have ever participated in a club meeting [AOR: 1.85; CI: 1.05, 3.24] were more likely to screen for breast cancer.

**Conclusion:**

The significant predictors of breast cancer screening were cervical cancer screening status, formal education, and participation in club meetings. Given that the prevalence of breast cancer screening among the older adult women in Ghana is low, we recommend that policies and programs dedicated to encouraging women to screen for breast cancer should aim at giving women the opportunity to obtain higher formal education, encouraging women to be actively involved in club meetings and to intensify efforts to encourage women to screen for breast cancer.

## 1 Introduction

Breast cancer screening is generally low in Ghana. Screening, a WHO-recommended cancer prevention and control measure, is simple, sustainable and cost-effective [1]. Screening helps in early detection of the disease and to reduce mortality. The absence of effective screening and treatment regimens largely contribute to low survival rates in low-and middle-income countries (LMICs) [2, 3]. Breast cancer is mostly diagnosed at the advanced stage in many LMICs including Ghana as a result of lack of screening for early detection [3, 4, 5], inadequately trained oncologist [3, 4, 5], inadequate knowledge about breast cancer among women [6], negative socio-cultural beliefs about cancers [6], and poor health infrastructure [3, 4]. Compared to the developed countries where there are routine cancer screening programs, many LMICs do not have such effective screening programs [4]. Specifically, there is no national screening program for cancers in Ghana [4].

Breast cancer is the commonly diagnosed cancer among women globally and fast-rising in LMICs [7]. The global incidence and mortality rates for breast cancer were around 2.1 million (11.6%) and 627,000 (6.6%) in 2018, respectively [7]. By 2024, about 19.5 million women are projected to be newly diagnosed with breast cancer, with over 55% to come from LMICs [8]. Breast cancer is more prevalent among older women compared to their younger counterparts [2]. Generally, older adults have a declining immune system, physical weakness, and prolonged exposure to multiple risk factors which may contribute to their susceptibility to non-communicable diseases including cancers. Globally, gender also is a key predisposing factor for all types of cancers, with women having a higher likelihood than men [7]. Studies have also found that a lack of participation in social groups is associated with a lower likelihood of breast cancer screening among women [9, 10, 11, 12, 13, 14, 15].

Although breast cancer and its cognate studies have received scholarly attention in Ghana [16, 17, 18], there is a paucity of current studies on factors that predict breast cancer screening among older adult women using nationally representative data. The present study addressed this gap by estimating the prevalence of and identifying the factors that predict breast cancer screening among older adult women in Ghana.

## 2 Methods

### 2.1 Study design

This is a cross-sectional study with secondary data from the 2014/2015 (wave II) Study on global AGEing and adult health (SAGE). The SAGE employed a multi-stage sampling design. It used random sampling to select clusters of sampling units and subsequently selected households using systematic sampling. Adults in selected households who consented to participate were enrolled and their sociodemographic data, as well as other health information, obtained.

### 2.2 Study sample

Complete data on 2,032 women 50 years and older constituted the study sample. The study sample selection process is documented in Table 1.

**Table 1:** The study sample selection process.

The breakdown			Total
Cases (participants) in the data set	4,735		
Participants less than 50 years	1,160	Excluded	3,575
Male participants 50 + years	1472	Excluded	2103
Cases with incomplete data	71	Excluded	2032
**Study Sample**			**2,032 women 50 years and older**

### 2.3 Outcome variable

The outcome variable was breast cancer screening by mammography. The respondents were asked if an x-ray of their breasts were ever taken to detect breast cancer at an early stage. Those who indicated yes were value labelled as “1” and all others as “0.”

### 2.4 Predictor variables

The predictor variables include cervical cancer screening through PAP smear test, age, education, marital status, difficulty caring for self, perceived sufficiency of money for basic needs, public meeting participation, club meeting participation, hosting friends at home, religious service participation, and locality of residence. Regarding the cervical cancer screening variable, participants were initially asked how long ago they have had cervix examination through a PAP smear if ever. All those who never had cervix examination, as well as those who had it but not by a PAP smear test, were value labelled as “0: No” and those who had cervix examination by a PAP smear test were value labelled as “1: Yes.” Table 2 contains detail information on the remaining variables: both how they were presented originally in the data set and how they were re-coded for the study.

**Table 2:** Recoding of the explanatory variables.

Variable in the original data set	Recoded for analysis
Age (continuous variable)	Age
	50–59 years
	60–69 years
	70–79 years
	80 + years
**Highest level of education**	**Education**
None	None
Less than primary	At most primary
Completed primary	
Completed secondary	Junior secondary
Completed high school	Senior secondary+
Completed college/university	
Completed post-grad	
**Marital status**	**Marital status**
Never married	Currently unmarried
Separated/divorced	
Widowed	
Currently married	Currently married
Cohabiting	
^**a**^**Difficulty caring for self**	**Difficulty caring for self**
None	None
Mild	Mild
Moderate	Moderate
Extreme	Extreme/severe
Severe	
^**b**^**Enough money**	**Perceived Sufficiency of Money for basic needs**
Not at all	Not at all
A little	A little
Moderately	Moderately
Mostly	Mostly/Completely
Completely	
^**c**^**Public meeting**	
Never	No
1/2 times per year	Yes
1/2 times per month	
1/2 times per week	
Daily	
^**d**^**Club**	**Club meeting participation**
Never	No
1/2 times per year	Yes
1/2 times per month	
1/2 times per week	
Daily	
^**e**^**Friends**	**Host Friends at home**
Never	No
1/2 times per year	Yes
1/2 times per month	
1/2 times per week	
Daily	
^**f**^**Religious services**	**Religious Service Participation**
Never	No
1/2 times per year	Yes
1/2 times per month	
1/2 times per week	
Daily	
**Urban/rural**	**Locality of residence**
Rural	Rural
Urban	Urban

Questions in the questionnaire: **a:** Overall in the last 30 days, how much difficulty did you have with self-care, such as bathing/washing or dressing yourself?; **b:** Do you have enough money to meet your needs?; **c:** How often in the last 12 months have you attended any public meeting in which there was discussion of local or school affairs?; **d:** How often in the last 12 months have you attended any group, club, society, union or organizational meeting?; **e:** How often in the last 12 months have you had friends over to your home?; **f:** attended religious services (not including weddings and funerals)?

### 2.5 Data preparations and analysis

Data was downloaded after permission was sought by the authors. Preliminary data cleaning was done in SPSS and final analysis was performed in the STATA-13 software. We employed a complex survey analysis design in STATA-13 to adjust for sampling design (sampling units, stratification, and population weights). We adopted this analytic method because the data used for the analysis were collected using a multistage sampling methodology. Thus, it is statistically prudent to account for the complex samples design and the population weight to ensure accurate estimates of confidence intervals and standard errors of predicted estimates [19, 20, 21]. We achieved this in STATA-13 by using the “svyset” command. After having accounted for the complex sample design, we performed summary statistics of the study variables, presenting both the unweighted and the weighted proportions. We discussed the weighted proportions because it is the true estimate of the study population.

After the summary statistics, we discovered a low prevalence of breast cancer screening in the sample, resulting in the sparsity of data. In such situations in epidemiological statistics, it is advised that the analysis should be done using exact logistic regression or the firth logistic method [22, 23, 24]. Due to the computational challenges associated with the use of exact logistic regression, we employed the firth logistic method for both bivariate and multivariate analyses. The advantage of the firth logistic regression method is that it decreases the small-sample bias inherent with generalized logistic models for rare outcomes [22, 23, 24]. Variables that were significant during bivariate analyses were included in the adjusted multivariable model. Statistical significance was pegged at p < 0.01 and p < 0.05.

### 2.6 Ethical clearance

The SAGE was approved by the World Health Organization's Ethical Review Board (reference number RPC149) and the Ethical and Protocol Review Committee, College of Health Sciences, University of Ghana, Accra, Ghana. All respondents gave written informed consent to be part of the study.

## 3 Results

### 3.1 Descriptive statistics of study variables

An estimated 4.5% and 1.7% of the older adult women in Ghana have ever undergone breast cancer and cervical cancer screening, respectively. The majority of them were within the age group of 50–59 years, have had no formal education, were currently unmarried, had no difficulty caring for themselves, never participated in public meetings, ever participated in club meetings, ever hosted friends at home, ever participated in religious services, and were residing in rural areas. Details of the summary statistics of the study variables are reported in Table 3.

**Table 3:** Complex sample summary statistics estimates of study variables. Factors that predict breast cancer screening.

Study Variables	WE [95% CI of WE]	UE
**Breast cancer screening**		

No	95.5% [93.5%, 96.8%]	1963 (96.6%)
Yes	4.5% [3.2%, 6.5%]	69 (3.4%)

**Cervical Cancer Screening**		

No	98.3% [97.5%, 98.8%]	1994 (98.13%)
Yes	1.7% [1.2%, 2.5%]	38 (1.87%)

**Age**		

50–59 years	48.2% [45.1%, 51.4%]	807 (39.7%)
60–69 years	25.9% [23.6%, 28.4%]	592 (29.1%)
70–79 years	16.7% [14.9%, 18.7%]	414 (20.4%)
80 + years	9.1% [7.8%, 10.6%]	219 (10.8%)

**Education**		
None	52.8% [49.1%, 56.5%]	1169 (57.5%)
At most Primary	27.9% [24.6%, 31.4%]	505 (24.9%)
Junior secondary	9.1% [7.2%, 11.6%]	160 (7.9%)
Senior secondary +	10.2% [8.4%, 12.4%]	198 (9.7%)

**Marital status**		

Currently unmarried	56.2% [53.0%, 59.4%]	1217 (59.88%)
Currently married	43.8% [40.6%, 47.0%]	815 (40.12%)

**Difficulty caring for self**		

None	74.3% [70.8%, 77.5%]	1539 (75.7)
Mild	17.7% [15.0%, 20.8%]	345 (17.0)
Moderate	6.3% [4.8%, 8.1%]	112 (5.5)
Extreme/severe	1.7% [1.2%, 2.4%]	36 (1.8)

**Perceived Sufficiency of Money for basic needs**		

Not at all	12.5% [10.3%, 15.0%]	257 (12.6)
A little	36.3% [33.3%, 39.4%]	795 (39.1)
Moderately	38.1% [35.0%, 41.3%]	786 (38.7)
Mostly/completely	13.1% [9.9%, 17.0%]	194 (9.5)

**Public meeting participation**		

No	62.6% [59.4%, 65.7%]	1303 (64.1)
Yes	37.4% [34.3%, 40.6%]	729 (35.9)

**Club meeting participation**		

No	44.1% [40.5%, 47.7%]	921 (45.3)
Yes	55.9% [52.3%, 59.5%]	1111 (54.7)

**Host friends at home**		

No	14.1% [11.7%, 16.9%]	251 (12.4)
Yes	85.9% [83.1%, 88.3%]	1781 (87.6)

**Religious Service Participation**		

No	12.5% [10.6%, 14.7%]	223 (11.0)
Yes	87.5% [85.3%, 89.4%]	1809 (89.0)

**Locality of residence**		

Rural	51.7% [49.1%, 54.1%]	1188 (58.5)
Urban	48.3% [45.9%, 50.9%]	844 (41.5)

WE: Weighted Estimate; UE: Unweighted Estimate; CI: Confidence Intervals

The bivariate analyses between the outcome and each predictor variable were conducted using the firth logistic regression. An older adult woman's cervical cancer screening status, formal education, participation in club meetings, and locality of residence were statistically significantly associated with breast cancer screening. These significant predictors were included in a multivariate firth logistic regression model. The model explained 14.4% of the variability in the outcome variable. Cervical cancer screening, having at least a primary level education, and having ever participated in a club meeting were significant predictors of breast cancer screening among older adult women in Ghana. The locality of residence lost its statistical significance in the multivariate model. Detail information of the odds ratios and the adjusted odds ratios of both the bivariate and multivariate models are reported in Table 4.

**Table 4:** Factors that predict breast cancer screening.

			Multivariable Model	
Study Variables	OR [95% CI]	P-Value	AOR [95% CI]	p-value
**Cervical cancer screening**				

No	1 (reference)			
Yes	15.89 [7.72, 32.71]	<0.001	13.29 [6.12, 28.84]	<0.001

**Age**				

50–59 years	1 (reference)			
60–69 years	1.19 [0.70, 2.01]	0.516		
70–79 years	0.47 [0.21, 1.05]	0.066		
80 + years	0.78 [0.33, 1.83]	0.564		

**Education**				
None	1 (reference)			
At most Primary	4.28 [2.28, 8.05]	<0.001	3.70 [1.94, 7.07]	<0.001
Junior secondary	5.73 [2.62, 12.52]	<0.001	4.02 [1.75, 9.21]	<0.001
Senior secondary +	6.73 [3.31, 13.71]	<0.001	4.57 [2.15, 9.71]	<0.001

**Marital status**				

Currently unmarried	1 (reference			
Currently married	1.09 [0.67, 1.77]	0.725		

**Difficulty caring for self**				

None	1 (reference)			
Mild	0.84 [0.43, 1.64]	0.603		
Moderate	0.11 [0.42, 2.96]	0.836		
Extreme/severe	0.37 [0.02, 6.05]	0.483		

**Perceived Sufficiency of Money for basic needs**				

Not at all	1 (reference)			
A little	1.15 [0.51, 2.62]	0.739		
Moderately	1.11 [0.49, 2.56]	0.791		
Mostly/completely	2.09 [0.82, 5.35]	0.123		

**Public meeting participation**				

No	1 (reference)			
Yes	1.57 [0.98, 2.54]	0.063		

**Club meeting participation**				

No	1 (reference)			
Yes	2.37 [1.38, 4.06]	0.002	1.85 [1.05, 3.24]	0.032

**Host friends at home**				

No	1 (reference)			
Yes	1.68 [0.70, 4.06]	0.247		

**Religious Service Participation**				

No	1 (reference)			
Yes	2.40 [0.81, 7.10]	0.113		

**Locality of residence**				

Rural	1 (reference)			
Urban	2.38 [1.46, 3.89]	0.001	1.65 [0.98, 2.77]	0.061

*Fit statistics of Multivariable Model*				

Wald χ^2^ (4)			81.12	
P-value			0.000	
Penalized log-likelihood			-251.240	
McFadden R^2^			0.144	

OR: Odds Ratio; AOR: Adjusted Odds Ratio

## 4 Discussion

The objective of the study was to estimate the prevalence and the predictors of breast cancer screening among older adult women in Ghana. The significant predictors were cervical cancer screening status, formal education, and participation in club meetings.

We found that breast cancer screening among older adult Ghanaian women was generally low (4.5%). This finding is not limited to older adult Ghanaian women only because anecdotal evidence suggests that the situation of the low prevalence of breast cancer screening is true for the general female population in Ghana. Literature reviews of studies from countries with similar economic and political conditions akin to Ghana have also reported that the uptake of breast cancer screening among women is unfortunately low [25, 26].

We found that older adult women who have ever been screened for cervical cancer had greater odds of ever screening for breast cancer. Women who have undergone cervical cancer screening may have possessed adequate knowledge about cancers and accepted that screening is the best strategy for early detection and treatment. Thus, at the time of undergoing a cervical cancer screening, the older adult women may have requested to be screened for breast cancer or may have been persuaded by the physician to equally screen for breast cancer.

Our study revealed that older adult women who have at least a primary education had higher odds of screening for breast cancer. The women who have had some form of formal education may have known and understood the implications of screening for cancer, resulting in their likelihood to undergo the screening. Our findings confirm the results of other studies from developing countries [27, 28]. These studies revealed that with an increase in each level of formal education, women were more likely to screen for breast cancer [27, 28].

We found that women who participated in club meetings were more likely to undergo breast cancer screening. In Ghana, anecdotal evidence suggests that many clubs create opportunities for members to be exposed to health promotion activities; this may even be more common among women-centred clubs. These health promotion activities sometimes involve the inviting of public health experts to educate members on the health implications of cancer and provide breast cancer screening for female members. Studies have also reported similar findings to ours [9]. For instance, a study from Malaysia revealed that women who belong to social support groups were more likely to have undergone breast cancer screening [9]. Additionally, studies from Brazil [11], Sweden [14], the 10.13039/100011408USA [13], Argentina [10], and Denmark [12] are in keeping with our finding that being part of a social support group increases women's chances to undergo breast cancer screening.

In the bivariate analysis, urban residency was found to be associated with higher odds of breast cancer screening among older adult women. This characteristic of the women, however, became statistically nonsignificant after controlling for other predictors. Although we found no relationship between place of residence and breast cancer screening in a multivariate model, a systematic review of 19 papers on breast cancer screening among women in China suggested that living in urban areas positively predict participation in breast cancer screening [15].

Our study has the following as limitations and strength. The data for our study was based on cross-sectional survey design, limiting the interpretation of our odds ratios to mere associations and not causal. Given that the prevalence in the outcome of interest is rare resulting in a separation or sparsity in the data, we had to employ the firth logistic regression to handle this challenge, which prevented us from accounting for the complex sample design of the dataset during the bivariate and multivariate logistic regression. One strength of the study is that we accounted for the complex sample design when estimating the prevalence of breast cancer screening among older adult women in Ghana.

## 5 Conclusion

We sought to estimate the prevalence of breast cancer and its predictors among older adult women in Ghana. We found that about 4.5% of older adult women in Ghana have undergone breast cancer screening. The significant predictors of breast cancer screening were cervical cancer screening status, formal education, and participation in club meetings. Given that the prevalence of breast cancer screening among the older adult women in Ghana is very low, we recommend that policies and programs dedicated to encouraging women to screen for breast cancer should aim at giving women the opportunity to obtain higher formal education, encouraging women to be actively involved in club meetings and to intensify efforts to encourage women to screen for breast cancer.

## Data availability statement

The 2014/15 SAGE data used to support the findings of this study may be released upon application to the WHO Multi-Country Studies Data Archive, who can be contacted at sagesurvey@who.int.

## Declarations

### Author contribution statement

Akosua F. Agyemang, Agnes Naki Tei-Muno, Veronica Millicent Dzomeku and Anna Boakyewaa Bentil: Conceived and designed the experiments; Wrote the paper.

Henry Ofori Duah and Precious Adade Duodu: Analyzed and interpreted the data; Wrote the paper.

Pascal Agbadi: Analyzed and interpreted the data; Contributed reagents, materials, analysis tools or data; Wrote the paper.

Emmanuel Kweku Nakua: Contributed reagents, materials, analysis tools or data; Wrote the paper.

### Funding statement

This research did not receive any specific grant from funding agencies in the public, commercial, or not-for-profit sectors.

### Competing interest statement

The authors declare no conflict of interest.

### Additional information

No additional information is available for this paper.
